# Effect of interleukin-8 receptor B (IL8RB) rs1126579 C>T variation on the risk to cancer

**DOI:** 10.1080/21655979.2021.1947442

**Published:** 2021-07-08

**Authors:** Lihong Zhu, Bowen Tang, Ze Zhang, Shuzhang Wei, Zhiwei Lv, Yujuan Zhang, Minlie Yang

**Affiliations:** a Department of Burn, Affiliated Hospital of Jiangnan University, Wuxi, China; b Department of Urology, The Affiliated Changzhou No.2 People’s Hospital of Nanjing Medical University, Changzhou, China; c Department of Operation Theatre, The Affiliated Changzhou No.2 People’s Hospital of Nanjing Medical University, Changzhou, China; d Department of Plastic Surgery, Affiliated Hospital of Jiangnan University, Wuxi, China

**Keywords:** Cancer, *IL8RB*, polymorphism, TCGA

## Abstract

Chemokines are a type of cytokine that participate in the migration of macrophages and monocytes to inflammatory cells. In particular, CXC chemokines are involved in the development of many cancers. Evidence for the association between *interleukin-8 receptor B* (*IL8RB*) rs1126579 C > T variation and cancer risk remains contradictory. Here, we utilized a comprehensive analysis containing odds ratios (ORs), regression, and *in silico* tools to evaluate the effect of *IL8RB* polymorphism on cancer risk. We further employed Gene set enrichment analysis combined with ELISA to evaluate the *IL8RB* expression in patients with prostate cancer (PRAD). A total of 5,187 cancer cases and 6,691 controls were included in the present analysis. Individuals with the TT genotype were associated with an increased risk of cancer compared to those with the TC+CC genotype. In a subgroup analysis by type of cancer, individuals with the TT genotype had a 39% increased risk of urinary cancer compared to those with the CC genotype. A subgroup analysis by ethnicity showed that Asians carrying the TC genotype had a 26% lower risk of cancer than those carrying the CC genotype. We found that the expression of *IL8RB* was down-regulated in PRAD. Compared to that in PRAD subjects carrying the CC genotype, the expression of *IL8RB* was decreased in patients with the TT+TC genotype. In conclusion, the *IL8RB* rs1126579 C > T variation may be associated with cancer risk, especially in Asian populations and patients with PRAD.

## Graphical abstarct

**Figure:**
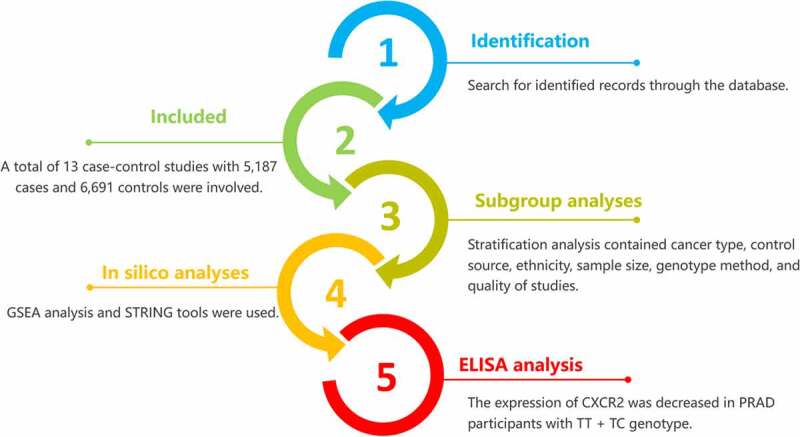

## Introduction

Cancer remains a major threat to human health worldwide[1]. In Western countries, the incidence and mortality of malignant tumors are growing, and the situation is similar in developing countries [2]. In 2020, the United States was expected to have 1,806,590 new cancer patients and 606,520 cancer-related deaths [3]. In Australia, the annual incidence of cancer was 31.7 per 100,000 people and the mortality rate was 4.1 in 100,000 from 2000 to 2009 [4]. The main reasons for the low survival rate of most malignant tumors are late diagnosis of the tumor at an advanced stage, metastasis, and resistance to treatment [5]. At present, specific markers for accurate diagnosis of many malignant tumors have not been identified. Therefore, it is necessary to develop some specific molecular markers to predict the prognosis of cancer patients and to provide effective targets for the treatment of these patients [6].

Chemokines belong to a large class of small protein molecules that play a vital role in many cellular activities, including cell recruitment and migration [7,8]. Previous researchers shown evidence that CXC chemokines and receptors can participate in a variety of functional activities including embryogenesis, angiogenesis, migration of leukocytes, and metastasis of malignancies [9–11]. Interleukin-8 (IL-8, CXCL8) is a small molecular basic protein that belongs to the ELR+-CXC chemokine subgroup. CXC chemokine receptor-2 (CXCR2) is a crucial IL-8 receptor and mediates angiogenesis induced by ELR+-CXC chemokines [12]. CXCR2, encoded by the *IL8RB* gene, is a seven-transmembrane G protein-coupled receptor (GPCR) that exists on the cell membrane of endothelial and cancer cells. Previous research revealed that the biological effect of IL-8 is mediated through the binding of CXCR1 or CXCR2 to IL-8 [13]. Subsequent studies demonstrated that CXCR2, but not CXCR1, is the crucial functional chemokine receptor that participates in angiogenesis induced by the chemokine and chemotaxis activity of endothelial cells [14–16]. The binding of CXCR2 and IL-8 can promote a series of tumor cell activities including proliferation, angiogenesis, and invasion [17,18]. The high affinity of CXCR2 to chemokines has been shown to be associated with the prognosis of patients with many cancers, including glioblastoma, colon cancer, lung cancer, hepatocellular carcinoma, and pancreatic cancer [19–23].

Genetic polymorphisms of *IL8RB* may directly influence the development of malignant tumors by inducing tumor angiogenesis and the immune response pathway [24,25]. Some studies revealed that expression of *IL8RB* is a potential adverse prognostic marker for individuals with non-metastatic renal clear cell carcinoma after nephrectomy [26]. However, several studies in other tumors failed to demonstrate a positive correlation between *IL8RB* and the recurrence and survival of patients [27,28]. The *IL8RB* rs1126579 C > T variation has been evaluated in rectal, prostate, stomach, bladder, esophageal, breast, colon, and lung cancer. Nevertheless, the relationship between this polymorphism and cancer risk remains incomprehensive. The aim of the current study was to comprehensively assess the association between *IL8RB* rs1126579 C > T variation and cancer risk based on all eligible case-control studies [29–38]. In addition, we used *in silico* analysis to evaluate the expression of *IL8RB* in prostate, bladder, breast, and lung cancer. We also investigated the correlation between the expression of *IL8RB* and the N stages of these patients. Moreover, we employed Gene Set Enrichment Analysis (GSEA) to investigate the expression of *IL8RB* in prostate cancer (PRAD), and ELISA to verify the findings in patients recruited from our centers.

## Materials and methods

### Search strategy

A database search was conducted according to the Embase, National Center for Biotechnology Information (NCBI) database, Chinese Wanfang, and Google Scholar. The following keywords were used: (‘rs1126579ʹ OR ‘*interleukin-8 receptor B*’ OR ‘*IL8RB*’) AND (‘cancer’ OR ‘tumor’ OR ‘carcinoma’) AND (‘mutation’ OR ‘variation’ OR ‘Single Nucleotide Polymorphism’ OR ‘SNP’ OR ‘mutant’). The last search update was 1 March 2021. We also searched the references and supplementary information of published articles to elevate the number of included studies.

### Inclusion and exclusion criteria

An appropriate study could be included in the current analysis if it met the following criteria: (a) case-control studies on the association between *IL8RB* rs1126579 C > T variation and susceptibility to cancer; (b) containing enough genotype data to measure odds ratios (ORs); and (c) manuscripts with available full text. The exclusion criteria were: (a) no available data in control; (b) insufficient information to assess the ORs; and/or (c) not related to *IL8RB* rs1126579 C > T variation and cancer risk.

### Data extraction

Data was classified according to the following characteristics: name of the author, year of publication, origin of patients, type of cancer, ethnicity of population, source of control, genetic data regarding *IL8RB* rs1126579 C > T variation, *P* value of Hardy-Weinberg equilibrium (HWE) in control, and method of genotyping. Cancers of different systems were divided into separated subgroups. The urinary cancer subgroup included prostate and bladder cancer. The digestive system cancer subgroup involved gastric, esophageal, colon, and rectal cancer. One study addressed to Kaposi’s sarcoma, and was defined as ‘other cancer’.

### Statistical analyses

Odds ratios (ORs) and 95% confidence intervals (CIs) were used to evaluate the strength of the correlation between *IL8RB* rs1126579 C > T variation and susceptibility to cancer. We employed five genetic models to evaluate the overall ORs of the rs1126579 C > T variant: allelic comparison (T allele vs. C allele), heterozygous (TC vs. CC), homozygous (TT vs. CC), dominant genetic model (TT + TC vs. CC), and recessive comparison (TT vs. TC + CC). The heterogeneity of the included studies was measured using a *Q* statistic test. If the *P*-value of heterogeneity (*P*_heterogeneity_) was < 0.05, a random effects calculation was adopted (DerSimonian and Laird) [39]. Conversely, a fixed effects method was selected (Mantel–Haenszel) if the *P*_heterogeneity_ was > 0.05 [40]. The *P* value of HWE (*P*_HWE_) was evaluated using Fisher’s exact test. Studies with a *P*_HWE_ > 0.05 were defined as high-quality groups. Otherwise, studies were defined as low-quality groups. If a study had a sample size greater than 1000, it was classified as a large sample group. Subgroup analysis contained the type of cancer, source of control, ethnicity, sample size, and quality of studies. Sensitivity analysis of *IL8RB* rs1126579 C > T variation was conducted by excluding every single study in turn. Publication bias was measured using Begg’s and Egger’s tests. *P* > 0.05 indicates that there is no evidence of publication bias among studies. Additionally, we used regression analysis to estimate the functional relationship of the log OR with the study characteristics. All the statistical analyses were performed using STATA software (v11.0, Stata Company, College Station, TX, USA).

### In silico and ELISA analysis

The minor allele frequencies (MAFs) in various populations were assessed using the Single Nucleotide Polymorphism database (dbSNP) of the NCBI repository (https://www.ncbi.nlm.nih.gov/snp). The gene expression profiles of *IL8RB* were investigated using the Gene Expression Profiling Interactive Analysis (GEPIA, http://gepia.cancer-pku.cn/index.html) and TNMplot databases (https://www.tnmplot.com/). Gene-gene interaction and expression of *IL8RB* in different populations were assessed using the Ualcan database (http://ualcan.path.uab.edu/analysis.html). The protein-protein correlation of CXCR2 was measured using the STRING online server (https://string-db.org/cgi/input.pl). The GSEA of the transcriptomes in the PRAD samples was determined using GSEA software (version 4.1.0, http://software.broadinstitute.org/gsea/index.jsp), a joint project produced by UC San Diego and the Broad Institute [41]. The immune pattern and landscape distribution between the high and low expression subtypes were investigated using the CIBERSORT computational method. We adopted the CIBERSORT algorithm to measure the proportions of tumor-infiltrating immune cells (TICs) in the PRAD samples [42]. Samples with a *P*-value less than 0.05 were chosen for follow-up analyses. ELISA analysis, according to the manufacturer’s instructions (CUSABIO Co., Ltd), was conducted based on samples from PRAD volunteers recruited in our centers [43,44]. A total of 220 needle biopsy-confirmed PRAD patients (by) were included from the Affiliated Changzhou No.2 People’s Hospital of Nanjing Medical University and the Affiliated Hospital of Jiangnan University. Every enrolled patient provided 2 mL of peripheral blood after signing the informed consent form. We then used ELISA to detect the serum expression of *IL8RB*. The above study was approved by the Ethics Committee of our hospitals.

## Results

In the current study, we used ORs, 95 CIs, and regression analysis to comprehensively assess the association between *IL8RB* rs1126579 C > T variation and cancer risk based on all eligible case-control studies. Moreover, we used *in silico* analysis, GSEA, and ELISA to explore the expression of *IL8RB*.

### Characteristics of studies

**Figure 1.**
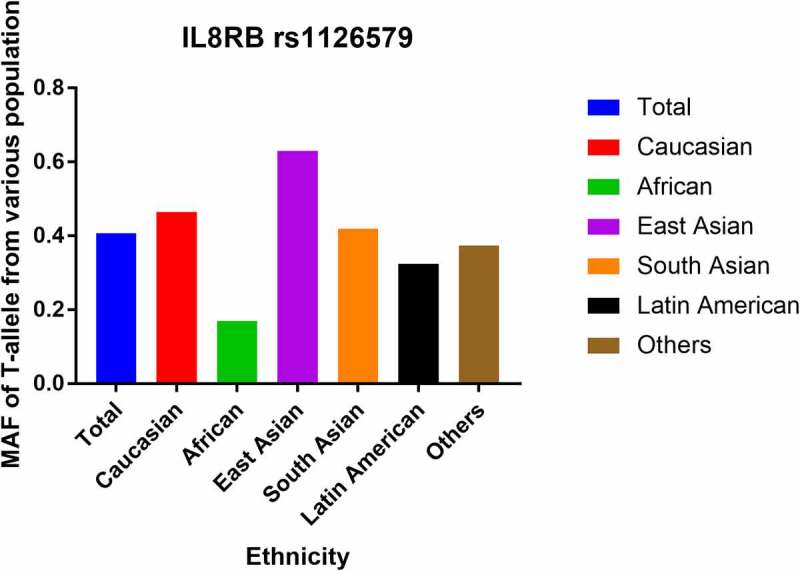
Minor allele frequencies of *IL8RB* rs1126579 C > T variation in various races

In total, 13 case-control studies with 5,187 cases and 6,691 controls were involved in the current analysis (Table 1). In the stratified analysis by cancer type, four studies were based on digestive cancer, and three studies each were based on urinary system tumors and lung cancer, respectively. Two studies focused on breast cancer, and one study was on Kaposi’s sarcoma and was classified as ‘other cancer’. There were six separate studies on Caucasians and Asians in the stratified analysis by ethnicity. An additional study was on an African population. In the subgroup analysis by control source, nine population-based (PB) and four hospital-based (HB) studies were included. The stratification analysis by genotyping method revealed seven studies utilizing the polymerase chain reaction (PCR) method, whereas the rest of the studies used either the Golden Gate method, Taqman assay, or iPLEX Gold method. In the stratified analysis by the quality of study, there were 10 high-quality and 3 low-quality case-control studies. In the subgroup analysis by the size of the population, there were 10 small size studies and 3 large size studies. In addition, the MAFs of *IL8RB* rs1126579 C > T polymorphism in various populations were evaluated by dbSNP. The MAFs for the rs1126579 C/T variant were: Africans, 0.169; Latin Americans, 0.325; Caucasian, 0.465; total population, 0.407; South Asians, 0.420; and East Asians, 0.630 (Figure 1).

**Table 1.** Study characteristics of *IL8RB* rs1126579 C > T variation in the present analysis

Author	Year	Origin	Cancer	Ethnicity	Source	Case	Control	Case			Control			HWE	Method
rs1126579 C/T								TT	TC	CC	TT	TC	CC		
Savage	2004	China	Gastric cancer	Asian	Population-based	89	415	11	22	56	59	154	202	0.001	PCR
Savage	2004	China	Esophageal cancer	Asian	Population-based	126	415	20	45	61	59	154	202	0.001	PCR
Brown	2006	Italy	Kaposi sarcoma	Caucasian	Population-based	133	172	14	40	79	16	69	87	0.666	Taqman
Lee	2007	China	Lung cancer	Asian	Population-based	115	107	55	47	13	44	51	12	0.628	PCR
Sarvestani	2007	Iran	Breast cancer	Asian	Hospital-based	218	261	27	85	106	27	114	120	0.992	PCR
Andrew	2009	USA	Bladder cancer	Caucasian	Population-based	589	863	250	255	84	299	414	150	0.745	GoldenGate
Snoussi	2010	Tunisia	Breast cancer	African	Hospital-based	409	301	47	167	195	18	128	155	0.207	PCR
Bondurant	2013	USA	Colon cancer	Caucasian	Population-based	1554	1956	343	794	417	411	1009	536	0.112	GoldenGate
Bondurant	2013	USA	Rectal cancer	Caucasian	Population-based	752	959	201	359	192	199	470	290	0.736	GoldenGate
Singh	2014	India	Bladder cancer	Asian	Hospital-based	200	200	15	73	112	14	90	96	0.247	PCR
Ryan	2015	USA	Lung cancer	Caucasian	Population-based	443	474	90	215	138	115	238	121	0.924	iPlexGold assay
Ryan	2015	Japan	Lung cancer	Asian	Population-based	384	383	170	160	54	178	170	35	0.537	Taqman
Franza	2017	Brazil	Prostate cancer	Caucasian	Hospital-based	175	185	25	133	17	28	131	26	<0.001	PCR

### Main results

**Figure 2.**
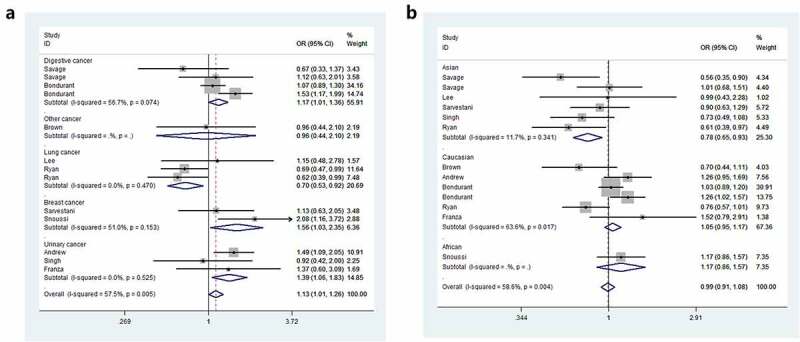
Forest plot of the correlation between *IL8RB* rs1126579 C > T variation and risk of cancer in stratified analysis by cancer type (Figure a) and ethnicity (Figure b)

**Figure 3.**
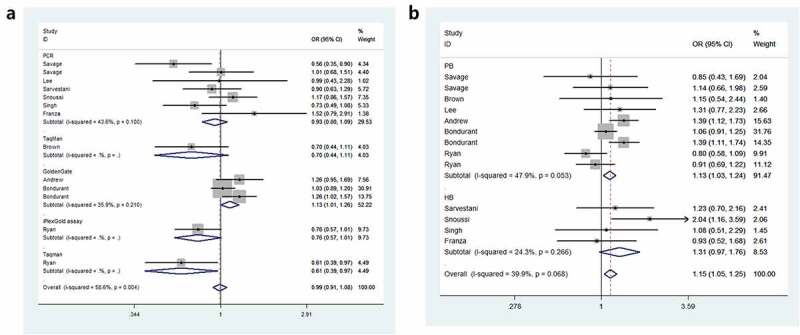
Forest plot of the relationship between *IL8RB* rs1126579 C > T variation and risk of cancer in subgroup analysis by method of genotyping (Figure a) and source of control (Figure B)

Odds ratios (ORs) and 95% Cls were employed to assess the relationship between *IL8RB* rs1126579 C > T polymorphism and cancer risk. Compared to those with the TC+CC genotype, individuals carrying the TT genotype were associated with an elevated risk of cancer (OR = 1.15, 95% CI = 1.05–1.25, *P* = 0.003, Table 2). In the subgroup analysis by cancer type, individuals with the TT genotype had a 39% increased risk of urinary cancer compared to those with the CC genotype (95% CI = 1.06–1.83, *P* = 0.018, Figure 2(a)). In the homozygous comparison, the *IL8RB* rs1126579 C > T polymorphism was also associated with increased breast and digestive cancer risk. Similar results were indicated in a recessive genetic model (breast cancer: OR = 1.60, 95% CI = 1.08–2.38, *P* = 0.020, digestive cancer: OR = 1.15, 95% CI = 1.02–1.31, *P* = 0.026). For lung cancer, individuals with the TT genotype had a 30% decreased risk compared to those with the CC genotype (95% CI = 0.53–0.92, *P* = 0.010). Similar findings were evident in the allelic contrast, heterozygous comparison, and dominant models. In the stratification analysis by ethnicity, Asian descendants carrying the TC genotype had a 26% lower risk of cancer than those carrying the CC genotype (95% CI = 0.61–0.89, *P* = 0.002). Similar findings were indicated in the dominant model (95% CI = 0.65–0.93, *P* = 0.005, Figure 2(b)). For Caucasian or African populations, no positive results were demonstrated. The subgroup analysis by method of genotyping revealed that *IL8RB* rs1126579 C > T variation was associated with augmented cancer risk, especially in studies performing the Golden Gate method (dominant model, 95% CI = 1.01–1.26, *P* = 0.041, Figure 3(a)). In the stratified analysis by control source, the TT genotype was associated with enhanced risk of cancer in PB studies using the recessive model (95% CI = 1.03–1.24, *P* = 0.010, Figure 3(b)). The stratification analysis by quality of study revealed that *IL8RB* rs1126579 C > T polymorphism was associated with elevated cancer risk in high-quality studies (homozygous model: 95% CI = 1.01–1.28, *P* = 0.028, recessive model: 95% CI = 1.16–1.27, *P* = 0.002). In the stratified analysis by sample size, different results were obtained from the large- and small-sample size studies. In the small-sample size studies, individuals carrying the TC genotype had a 19% attenuated risk compared to those carrying the CC genotype (95% CI = 0.71–0.93, *P* = 0.003). For the large-sample size studies, patients carrying the T-allele had a 16% augmented risk compared to those with the C-allele (95% CI = 1.01–1.33, *P* = 0.033).

**Table 2.** Stratified analysis of *IL8RB* rs1126579 C > T polymorphism on cancer susceptibility

Variables	N	Case/	OR (95%CI) *P*_heter_ *P*	OR (95%CI) *P*_heter_ *P*	OR (95%CI) *P*_heter_ *P*	OR (95%CI) *P*_heter_ *P*	OR (95%CI) *P*_heter_ *P*
		Control	T-allele vs. C-allele	TC vs. CC	TT vs. CC	TT+TC vs. CC	TT vs. TC+CC
**rs1126579 C/T**							
Total	13	5187/6691	1.01(0.91–1.12) 0.001 0.837	0.90(0.79–1.03) 0.044 0.134	1.09(0.89–1.33) 0.005 0.393	0.94(0.81–1.09) 0.004 0.414	1.15(1.05–1.25) 0.068 0.003
**Cancer Type**							
Digestive	4	2521/3745	1.03(0.86–1.23) 0.011 0.735	1.01(0.89–1.14) 0.060 0.900	1.17(1.01–1.36) 0.074 0.035	0.99(0.77–1.27) 0.020 0.931	1.15(1.02–1.31) 0.221 0.026
Lung	3	942/964	0.87(0.76–0.99) 0.301 0.033	0.74(0.58–0.95) 0.633 0.018	0.70(0.53–0.92) 0.470 0.010	0.73(0.58–0.92) 0.566 0.008	0.91(0.75–1.11) 0.282 0.342
Breast	2	627/562	1.13(0.95–1.35) 0.192 0.166	0.96(0.75–1.22) 0.415 0.711	1.56(1.03–2.35) 0.153 0.034	1.05(0.83–1.32) 0.278 0.684	1.60(1.08–2.38) 0.211 0.020
Urinary	3	964/1248	1.14(1.01–1.30) 0.054 0.033	0.99(0.79–1.25) 0.079 0.960	1.39(1.06–1.83) 0.153 0.018	1.08(0.71–1.66) 0.047 0.712	1.31(1.08–1.59) 0.398 0.007
Other	1	133/172	0.83(0.58–1.18) – 0.299	0.64(0.39–1.05) – 0.075	0.96(0.44–2.10) – 0.926	0.70(0.44–1.11) – 0.126	1.15(0.54–2.44) – 0.722
**Ethnicity**							
Asian	6	1132/1781	0.90(0.80–1.02) 0.320 0.090	0.74(0.61–0.89) 0.486 0.002	0.86(0.67–1.11) 0.495 0.253	0.78(0.65–0.93) 0.341 0.005	1.03(0.84–1.25) 0.812 0.791
Caucasian	6	3646/4609	1.05(0.92–1.21) 0.002 0.457	1.01(0.91–1.12) 0.110 0.851	1.15(0.89–1.49) 0.009 0.281	1.05(0.95–1.17) 0.017 0.301	1.13(0.94–1.37) 0.029 0.185
African	1	409/301	1.25(0.99–1.58) – 0.058	1.04(0.76–1.42) – 0.819	2.08(1.16–3.72) – 0.014	1.17(0.86–1.57) – 0.315	2.04(1.16–3.59) – 0.013
**Source**							
PB	9	4185/5744	1.00(0.88–1.13) <0.001 0.963	0.88(0.75–1.05) 0.036 0.150	1.02(0.81–1.29) 0.003 0.854	0.91(0.76–1.10) 0.003 0.342	1.13(1.03–1.24) 0.053 0.010
HB	4	1002/947	1.05(0.92–1.21) 0.199 0.443	0.93(0.76–1.13) 0.174 0.462	1.39(1.00–1.93) 0.338 0.052	0.99(0.82–1.20) 0.139 0.952	1.31(0.97–1.76) 0.266 0.078
**Method**							
PCR	7	1332/1884	1.02(0.91–1.14) 0.119 0.783	0.88(0.74–1.03) 0.160 0.118	1.19(0.92–1.53) 0.360 0.180	0.93(0.80–1.09) 0.100 0.384	1.22(0.98–1.52) 0.509 0.076
Taqman	2	517/555	0.84(0.70–1.01) 0.889 0.071	0.62(0.44–0.88) 0.897 0.007	0.70(0.47–1.04) 0.342 0.080	0.65(0.48–0.90) 0.693 0.010	0.94(0.72–1.23) 0.583 0.653
GoldenGate	3	2895/3778	1.16(1.01–1.33) 0.028 0.033	1.06(0.94–1.20) 0.631 0.323	1.26(1.10–1.45) 0.056 0.001	1.13(1.01–1.26) 0.210 0.041	1.22(1.09–1.37) 0.065 < 0.001
iPlexGold	1	443/474	0.83(0.69–0.99) – 0.040	0.79(0.58–1.08) – 0.135	0.69(0.47–0.99) – 0.045	0.76(0.57–1.01) – 0.059	0.80(0.58–1.09) – 0.152
**Quality**							
Low	3	390/1015	0.94(0.79–1.13) 0.113 0.532	0.90(0.51–1.60) 0.032 0.719	0.99(0.67–1.46) 0.383 0.969	0.92(0.54–1.56) 0.034 0.761	0.98(0.70–1.39) 0.791 0.926
High	10	4797/5676	1.06(1.00–1.12) 0.001 0.053	0.95(0.87–1.05) 0.111 0.310	1.14(1.01–1.28) 0.002 0.028	0.95(0.82–1.11) 0.010 0.535	1.16(1.06–1.27) 0.027 0.002
**Sample size**							
Small	10	2292/2913	0.93(0.86–1.02) 0.083 0.116	0.81(0.71–0.93) 0.193 0.003	0.92(0.77–1.11) 0.079 0.403	0.85(0.75–0.96) 0.083 0.010	1.02(0.88–1.18) 0.300 0.800
Large	3	2895/3778	1.16(1.01–1.33) 0.028 0.033	1.06(0.94–1.20) 0.631 0.323	1.26(1.10–1.45) 0.056 0.001	1.13(1.01–1.26) 0.210 0.041	1.22(1.09–1.37) 0.065 < 0.001

HB: Hospital based; N: Number of included studies; PCR: polymerase chain reaction; PB: Population based.

*P*_heter_: *P* value of heterogeneity test.

### In silico and ELISA analysis

**Figure 4.**
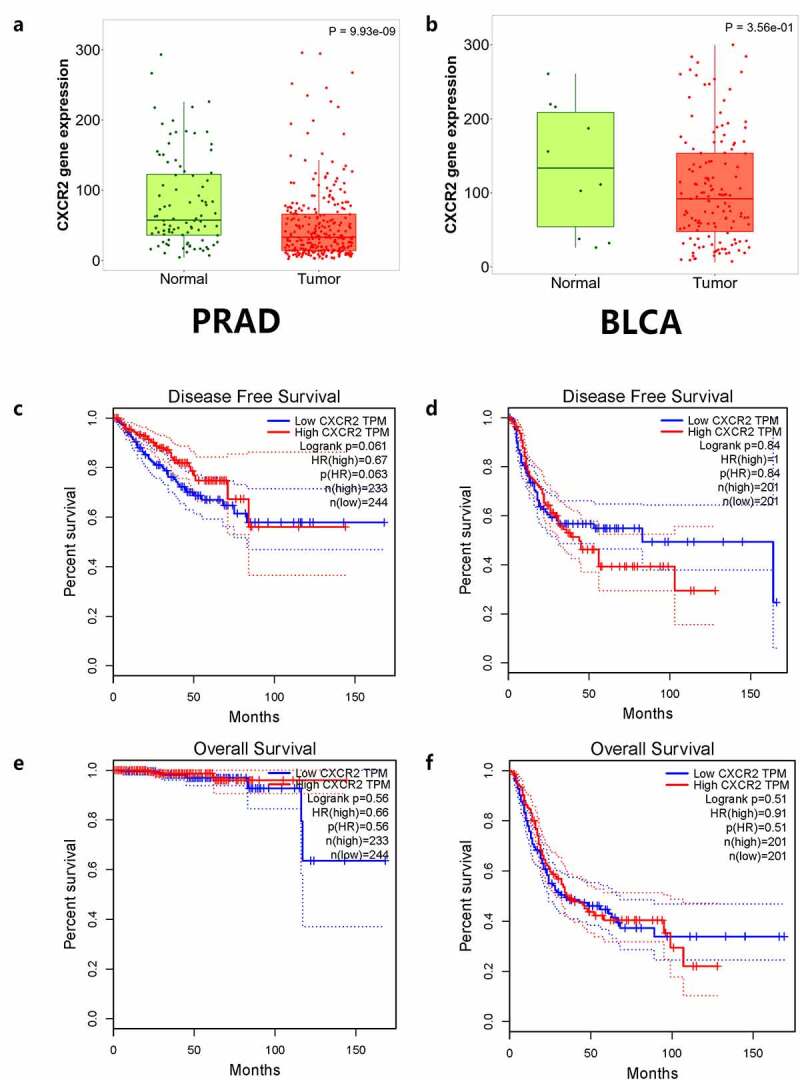
*In silico* analysis of *IL8RB* expression in prostate cancer (PRAD) and bladder cancer (BLCA) patients. Result from TNMplot database showed that the expression of *IL8RB* was both down-regulated in PRAD (Figure A) and BLCA (Figure B) subjects. Effect of *IL8RB* expression on PRAD patients’ disease-free survival (DFS) and overall survival (OS) time was described in Figure C and E. At the first 50 months, PRAD patients with low *IL8RB* expression may have a shorter DFS than the high expression group. The expression of *IL8RB* on BLCA participants’ DFS and OS time was described in Figure D and E. No obvious difference on the DFS and OS time was indicated between the low *IL8RB* expression and high expression group among BLCA subjects

**Figure 5.**
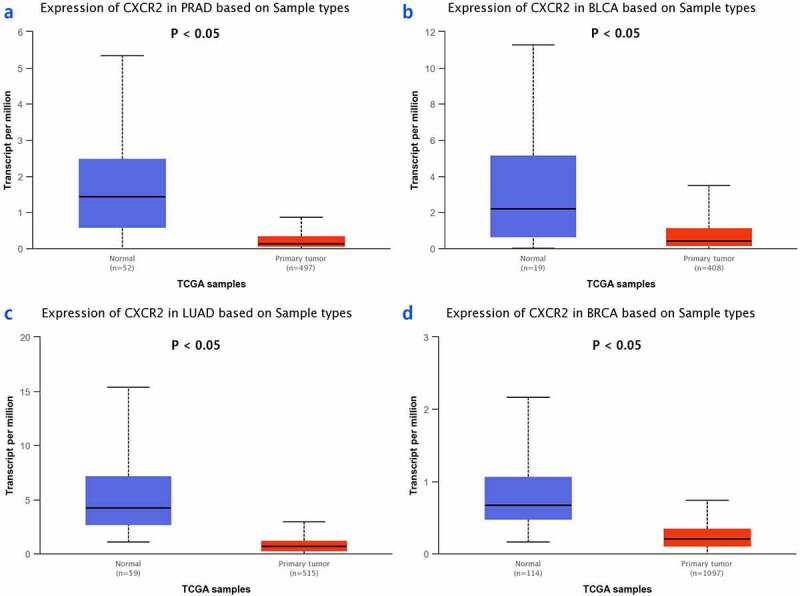
Expression of *IL8RB* in various types of cancer. Expression of *IL8RB* was down-regulated in both PRAD and BLCA patients (Figure A and B, *P* < 0.05). The expression of *IL8RB* was also down-regulated in lung cancer and breast cancer patients (Figure C and D, *P* < 0.05)

**Figure 6.**
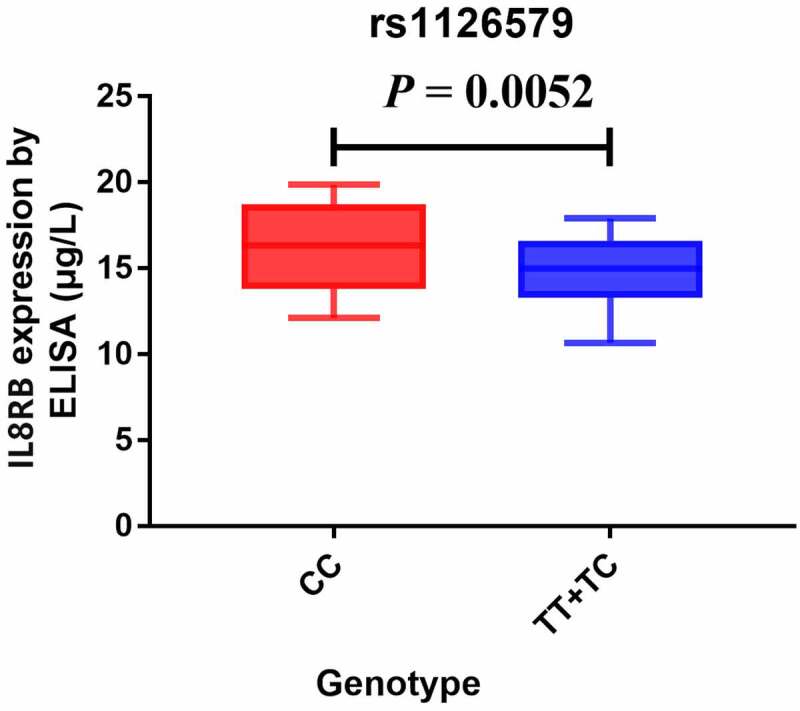
Analysis of serum expression of *IL8RB* in PRAD subjects by ELISA. The expression of *IL8RB* was decreased in PRAD participants with TT + TC genotype (*P* < 0.05)

**Figure 7.**
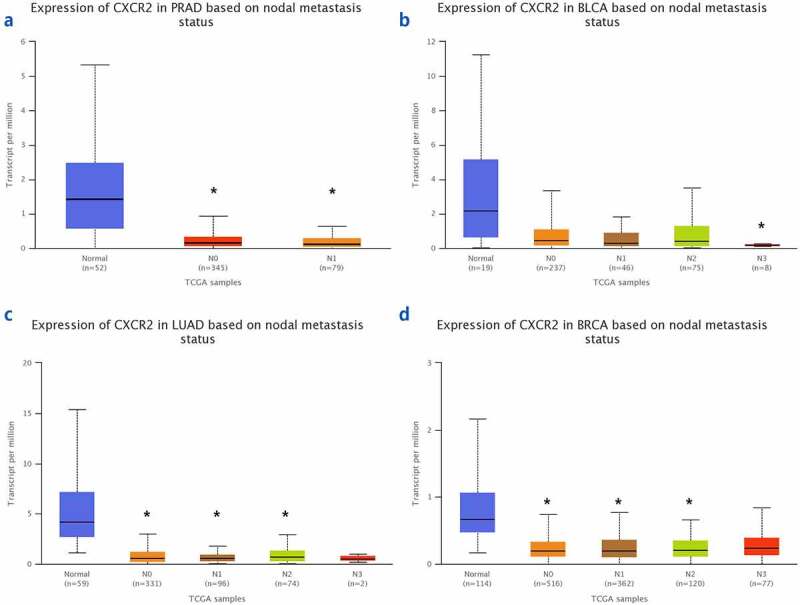
The correlation between the *IL8RB* expression and the N stages of cancer patients. For PRAD, the expression was decreased in both N0 and N1 patients than that in normal counterparts (*P* < 0.05, Figure A). For BLCA, the expression of *IL8RB* was only down-regulated in N3 patients (*P* < 0.05, Figure B). For lung cancer, the expression was attenuated in patients with N0, N1, and N2 stage (*P* < 0.05, Figure C). For breast cancer, the expression of *IL8RB* was also diminished in patients with N0, N1, and N2 stage (*P* < 0.05, Figure D)

The TNMplot database was used to evaluate the expression of *IL8RB* in PRAD and bladder cancer (BLCA) patients. The expression of *IL8RB* was down-regulated in both the PRAD (Figure 4(a), *p* < 0.05) and BLCA (Figure 4(b), *p* < 0.05) subjects. Effect of *IL8RB* expression on PRAD patients’ disease-free survival (DFS) and overall survival (OS) time was shown in Figure 4(c,e). In the first 50 months, PRAD patients with low *IL8RB* expression had a shorter DFS time than that in the high expression groups. The expression of *IL8RB* on BLCA participants’ DFS and OS time was described in Figure 4(d,e). No significant difference on the DFS and OS time was indicated between the low and high expression groups among BLCA subjects. We further used the Ualcan database to verify the expression of *IL8RB* in various types of cancer. Expression of *IL8RB* was down-regulated in both PRAD and BLCA (Figure 5(a,b), *p* < 0.05). Expression of *IL8RB* was also down-regulated in lung and breast cancer patients (Figure 5(c,d), *p* < 0.05). Furthermore, ELISA was employed to assess the serum expression of *IL8RB* in PRAD patients recruited from our hospitals. Compared with that in PRAD subjects carrying the CC genotype, the expression was decreased in patients with the TT + TC genotype (Figure 6, *P* < 0.05). Moreover, we investigated the correlation between *IL8RB* expression and the N stages of cancer patients. For PRAD, expression was decreased in both N0 and N1 patients compared to that in their normal counterparts (*P* < 0.05, Figure 7(a)). For BLCA, the expression of *IL8RB* was only down-regulated in N3 patients (*P* < 0.05, Figure 7(b)). For lung cancer, the expression was attenuated in patients with N0, N1, and N2 stage cancer (*P* < 0.05, Figure 7(c)). For breast cancer, the expression of *IL8RB* was also diminished in patients with N0, N1, and N2 stage cancer (*P* < 0.05, Figure 7(d)).

**Figure 8.**
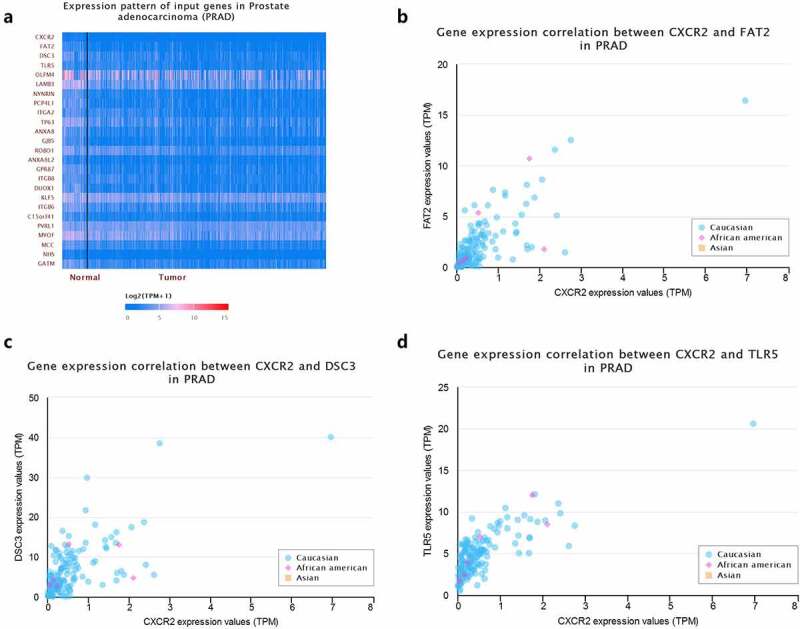
Gene-gene interaction of *IL8RB* in PRAD patients. Differential expressed genes between high *IL8RB* expression and low expression group was described in Figure A. The most correlated genes include *VSTM2L* (*V-set and transmembrane domain containing 2 like* gene, *P* < 0.05, R = −0.19, Fig. B), *CRISP3* (*cysteine rich secretory protein 3* gene, *P* < 0.05, R = −0.08, Fig. C), *DLX1* (*distal-less homeobox 1 gene, P* < 0.05, R = −0.29, Fig. D)

**Figure 9.**
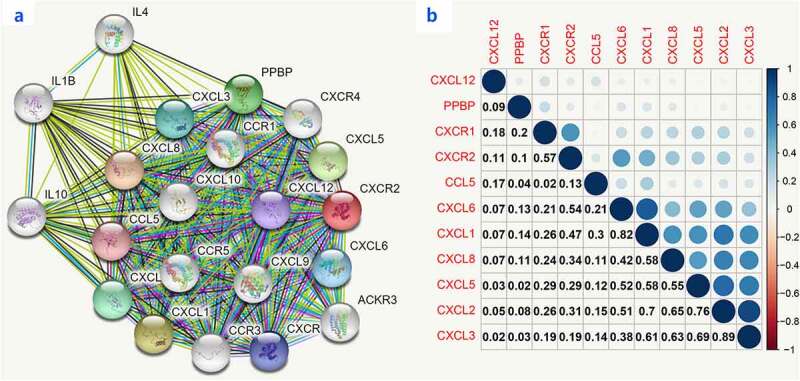
Crosstalk of CXCR2 protein assessed by the STRING tools. More than 20 proteins can participate in interacting with CXCR2 (Figure A). The correlations of the top ten proteins were described in Figure B

**Figure 10.**
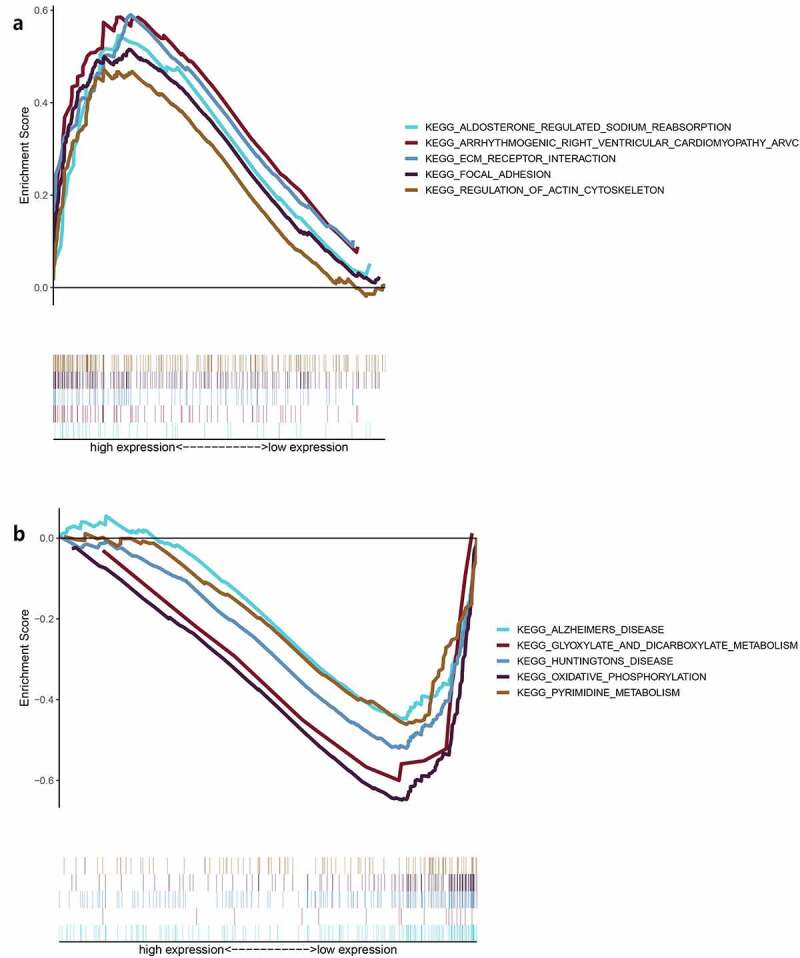
Gene Set Enrichment Analysis (GSEA) for samples with high *IL8RB* and low expression. Enriched gene set of *IL8RB* high expression samples in KEGG collection (Figure A). Each line represents a specific set of genes with a unique color. The up-regulated genes were on the left (close to the origin of the coordinates), while the down-regulated genes are on the right side of the x-axis. The gene set enriched in KEGG for samples with low *IL8RB* expression (Figure B)

**Figure 11.**
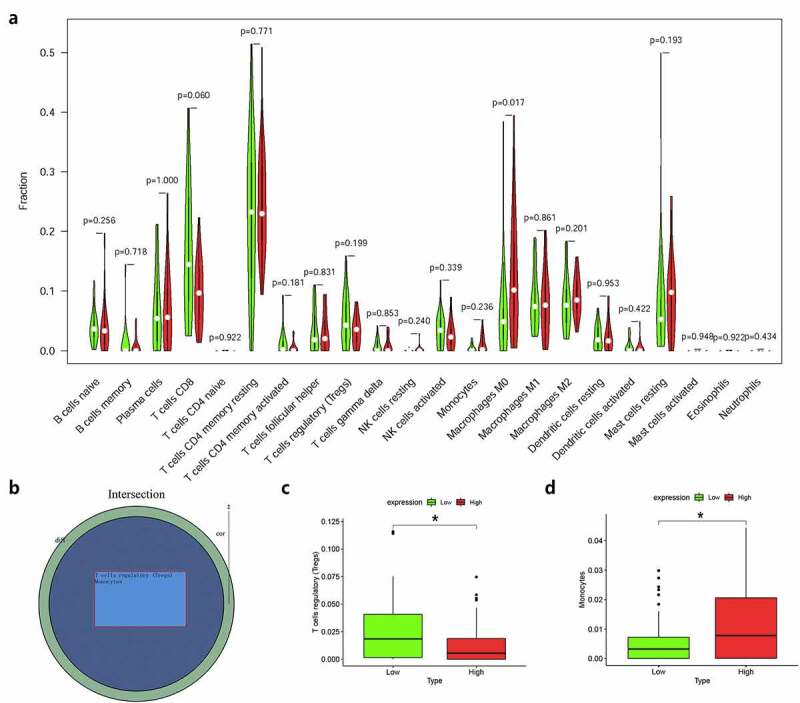
The relationship between the expression of *IL8RB* and the proportion of tumor-infiltrating immune cells (TICs). Violin plot displayed the difference of 22 kinds of immune cells with low or high expression of *IL8RB* to the median level of *IL8RB* expression in PRAD (Figure A and B). Compared with low *IL8RB* expression group, the proportion of T regulatory cells was significantly attenuated in high expression group (Fig. C). Meanwhile, the proportion of monocytes was relatively augmented in high expression group (Fig. D)

Moreover, we used R language to investigate the gene-gene correlations of *IL8RB*. More than 24 genes were involved in interactions with the *IL8RB* gene in PRAD (Figure 8(a)). The most correlated genes included *VSTM2L* (*V-set and transmembrane domain containing 2 like* gene, *P* < 0.05, R = −0.19, Figure 8(b)), *CRISP3* (*cysteine rich secretory protein 3* gene, *P* < 0.05, R = −0.08, Figure 8(c)), and *DLX1* (*distal-less homeobox 1* gene, *P* < 0.05, R = −0.29, Figure 8(d)). We further employed the STRING database to investigate the protein-protein correlation of CXCR2. More than 20 proteins could interact with the CXCR2 protein (Figure 9(a)). The correlations of the top ten proteins were described in Figure 9(b). In addition, we performed GSEA analysis to investigate the potential associated signaling pathways correlated with expression of *IL8RB*. As described in Figure 10(a), signaling pathways including aldosterone regulated sodium reabsorption, extracellular matrix (ECM) receptor, focal adhesion, and regulation of actin cytoskeleton were associated with high expression of *IL8RB*. Meanwhile, signaling pathways such as glyoxylate and dicarboxylate metabolism, oxidative phosphorylation, and pyrimidine metabolism were correlated with low expression of *IL8RB* (Figure 10(b)). Furthermore, we used the CIBERSORT computational method to evaluate the abundance profile of TICs in PRAD samples (Figure 11(a,b)). Compared with that in the low *IL8RB* expression group, the proportion of T regulatory cells was significantly attenuated in the high expression group (Figure 11(c)). Moreover, the proportion of monocytes was relatively augmented in the high expression group (Figure 11(d)).

### Sensitivity, regression analysis, and publication bias

**Figure 12.**
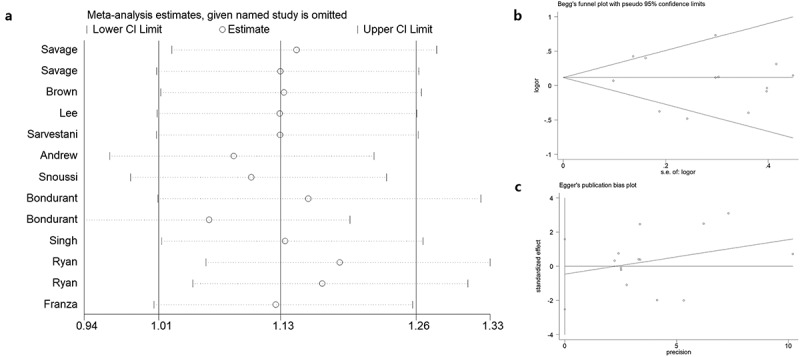
Publication bias of the current study assessed by sensitivity analysis, Begg’s funnel plot, and Egger’s test. Sensitivity analysis of *IL8RB* rs1126579 C > T variation showed that a single study would not have an impact on the significance of ORs (Figure A). Begg’s funnel (Figure B) and Egger’s plot (Figure C) analysis also indicated no evidence of publication bias

**Figure 13.**
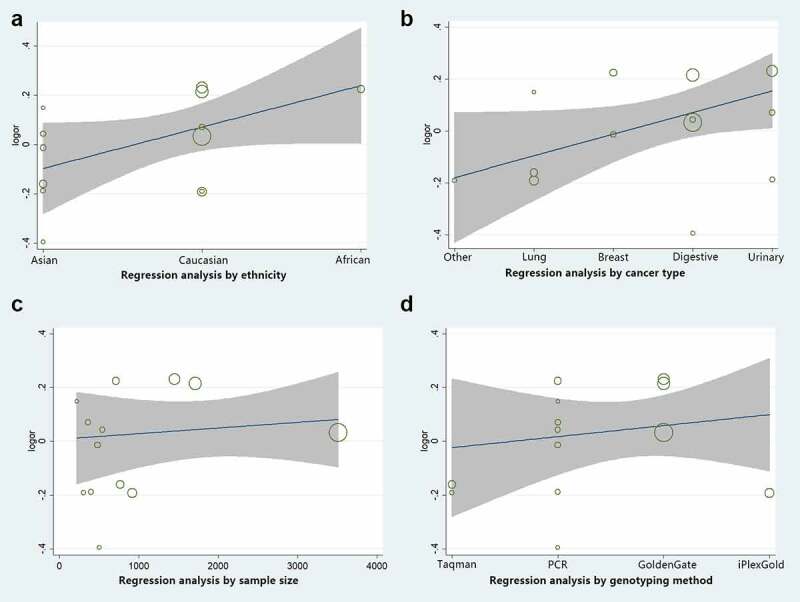
Regression analysis of log odds ratio versus subgroup analysis of study ethnicity, genotyping method, sample size. No evidence of heterogeneity among the studies was revealed (Fig. A, B, C, and D)

Sensitivity analysis was employed to reveal the effect of a single study on the overall ORs. Publication bias was assessed using Begg’s and Egger’s tests. As shown in Figure 12(a), no single study was found to have a significant impact on the ORs when assessing the *IL8RB* rs1126579 C > T variation. Begg’s (Figure 12(b), *P* > 0.05) and Egger’s tests (Figure 12(c), *P* > 0.05) also identified no evidence of publication bias in the studies of *IL8RB* polymorphism. Moreover, we adopted regression analysis to determine whether there was a significant correlation between the studied ethnicity, genotyping method, sample size, and combined OR. No evidence of heterogeneity among the studies was revealed (Figure 13(a-d)).

## Discussion

Cancer is a huge health problem worldwide. Although most cancer patients receive standard treatment including operation, radiotherapy, chemotherapy, or immunotherapy, not everyone can benefit from these strategies. Previous studies have shown evidence that expression of *IL8RB* is related to necrosis and development of several cancers [23,45,46]. Furthermore, expression of *IL8RB* can act as an autocrine or paracrine growth factor in the invasion and migration of cancer [47]. Genetic variants of *IL8RB* may affect the function of the protein by influencing gene expression. The correlation between *IL8RB* variations and cancer risk has been evaluated in previous studies [32–38]. However, the conclusions have been contradictory. Singh *et al*. assessed the *IL8RB* rs1126579 C > T variation in Indians and revealed that the T allele was associated with an increased risk of BLCA (*P* = 0.003, OR = 1.29) [36]. Another researcher assessed the *IL8RB* variant in two races (Caucasian and Asian) and observed that rs1126579 C > T variation was associated with a decreased risk of lung cancer [37]. A meta-analysis published in 2017 showed that *IL8RB* expression in cancer was related to poor prognosis of patients [47]. One year later, another meta-analysis demonstrated that *IL8RB* expression is a poor predictor for digestive cancer patients [48]. However, the above studies did not assess the correlation between *IL8RB* rs1126579 C > T variation and susceptibility to cancer. Therefore, we performed a comprehensive analysis based on 5,187 cancer cases and 6,691 controls from 13 case-control studies. We observed a positive association between *IL8RB* rs1126579 C > T mutation and cancer risk.

In the subgroup analysis by cancer type, we revealed that *IL8RB* rs1126579 C > T variation was correlated with an elevated risk of urinary and breast cancer, as well as cancer in the digestive system. For lung cancer, individuals with the TT genotype had a 30% decreased risk compared to those with the CC genotype. Our results are in line with those reported in previous studies [37]. In the stratification analysis by ethnicity, Asian individuals carrying the TC genotype had a 26% lower risk of cancer than those carrying the CC genotype. However, we observed no positive results in the Caucasian or African participants. The possible reason may be that the sample size of studies during the subgroup analysis was relatively small. However, there were some studies indicating that *IL8RB* rs1126579 C > T variation was associated with an elevated risk of breast cancer in African populations [34]. As described in the stratified analysis by sample size, the results of studies with a large sample size may be different from those with a small sample size. Therefore, further research with large sample sizes on *IL8RB* rs1126579 C > T polymorphism in African descendants is required in the future. Furthermore, *in silico* analysis was utilized to explore the expression of *IL8RB* in urinary cancer based on the race of patients. Expression of *IL8RB* was diminished in BLCA patients of Caucasian, African-American, and Asian descent. The expression was also mitigated in Caucasian and African-American PRAD patients. To verify the reliability of the results obtained from the online database, we used ELISA to detect the serum *IL8RB* expression in the pathologically confirmed PRAD patients recruited from our centers. It showed that *IL8RB* expression was attenuated in PRAD patients with the TT+TC genotype, which was consistent with the results of the present analysis. In addition, expression of *IL8RB* was down-regulated in several cancers including PRAD, BLCA, lung cancer, and breast cancer. For PRAD, the expression was decreased in both N0 and N1 patients. For BLCA, the expression of *IL8RB* was only down-regulated in N3 patients. For lung cancer, the expression was attenuated in patients with N0, N1, and N2 stage cancer. For breast cancer, the expression of *IL8RB* was also diminished in patients with N0, N1, and N2 stage cancer.

Previous study has adopted TCGA database to explore prognostic factors for testicular germ cell tumors [49]. In addition, researchers also used this database to identify a genomic lncRNA signature to provide guidance for the treatment of patients with BLCA [50]. In the present study, we employed the TCGA database to investigate signaling pathways associated with expression of *IL8RB*. Several signaling pathways, such as ECM receptor, focal adhesion, regulation of actin cytoskeleton, and aldosterone regulated sodium re-absorption, were associated with high *IL8RB* expression. Moreover, we used the CIBERSORT method to investigate the TIC abundance in PRAD samples between the high and low *IL8RB* expression groups. Compared with that in the low *IL8RB* expression group, the proportion of T regulatory cells was significantly attenuated in the high expression group. Meanwhile, the proportion of monocytes was relatively augmented in the high expression group. Besides, there are several limitations in the above analysis. First, the number of studies on African populations is fairly small. More studies of these populations with large sample sizes are required. Second, the sample size of case-control studies on *IL8RB* rs1126579 C > T variation remains insufficient. The number of studies for the subgroup analysis of cancer types was also insufficient, especially for PRAD, BLCA, and renal cell carcinoma. Third, we revealed that *IL8RB* rs1126579 C > T polymorphism may be related to an elevated risk of PRAD. Further research is still needed to ascertain whether this variant can affect the expression of *IL8RB* in PRAD. Since a single mutation cannot have a great impact on the occurrence and development of cancer, future studies on gene-gene or gene-environment interactions are still warranted.

## Conclusion

Taken together, the current study summarizes all eligible genetic data for association between *IL8RB* rs1126579 C > T variation and cancer risk. Our study revealed that the *IL8RB* rs1126579 C > T polymorphism is associated with an increased risk of urinary, breast, and digestive cancer, especially in individuals of Asian descent. *IL8RB* rs1126579 C > T variation may also be correlated with the risk of PRAD.

## Highlights

1. We used odds ratios, regression, and *in silico* analysis to assess the effect of *IL8RB* variation on cancer risk.
2. The current analysis showed that *IL8RB* rs1126579 C>T variation may be associated with risk of cancer.
3. Expression of *IL8RB* is decreased in PRAD patients.
4. GSEA showed that signaling pathways including ECM receptor and focal adhesion were associated with high expression of *IL8RB*.

## Data accessibility

All the data of the current study can be acquired from corresponding authors upon request.

## References

[1] Sung H, Ferlay J, Siegel RL, et al. Global cancer statistics 2020: GLOBOCAN estimates of incidence and mortality worldwide for 36 cancers in 185 countries. CA Cancer J Clin. 2021;71:209–249. 33538338 10.3322/caac.21660

[2] Miller KD, Fidler-Benaoudia M, Keegan TH, et al. Cancer statistics for adolescents and young adults, 2020. CA Cancer J Clin. 2020;70:443–459. 32940362 10.3322/caac.21637

[3] Siegel RL, Miller KD, Jemal A. Cancer statistics, 2020. CA Cancer J Clin. 2020;70:7–30. 31912902 10.3322/caac.21590

[4] Roder DM, Warr A, Patterson P, et al. Australian adolescents and young adults-trends in cancer incidence, mortality, and survival over three decades. J Adolesc Young Adult Oncol. 2018;7:326–338. 29373040 10.1089/jayao.2017.0095

[5] Wang X, Guo J, Yu P, et al. The roles of extracellular vesicles in the development, microenvironment, anticancer drug resistance, and therapy of head and neck squamous cell carcinoma. J Exp Clin Cancer Res. 2021;40:35. 33478586 10.1186/s13046-021-01840-x PMC7819156

[6] Jemal A, Thun MJ, Ries LA, et al. Annual report to the nation on the status of cancer, 1975-2005, featuring trends in lung cancer, tobacco use, and tobacco control. J Natl Cancer Inst. 2008;100:1672–1694. 19033571 10.1093/jnci/djn389 PMC2639291

[7] Vinader V, Afarinkia K. The emerging role of CXC chemokines and their receptors in cancer. Future Med Chem. 2012;4:853–867. 22571611 10.4155/fmc.12.48

[8] Bie Y, Ge W, Yang Z, et al. The crucial role of CXCL8 and its receptors in colorectal liver metastasis. Dis Markers. 2019;2019:8023460. 31827643 10.1155/2019/8023460 PMC6886345

[9] Mollica Poeta V, Massara M, Capucetti A, et al. Chemokines and chemokine receptors: new targets for cancer immunotherapy. Front Immunol. 2019;10:379. 30894861 10.3389/fimmu.2019.00379 PMC6414456

[10] Raffaghello L, Cocco C, Corrias MV, et al. Chemokines in neuroectodermal tumour progression and metastasis. Seminars in cancer biology 2009; 19:97–102. 19013246 10.1016/j.semcancer.2008.10.003

[11] Gonzalez-Aparicio M, Alfaro C. Influence of Interleukin-8 and Neutrophil Extracellular Trap (NET) formation in the tumor microenvironment: is there a pathogenic role? J Immunol Res. 2019;2019:6252138. 31093511 10.1155/2019/6252138 PMC6481028

[12] Belperio JA, Keane MP, Arenberg DA, et al. CXC chemokines in angiogenesis. J Leukoc Biol. 2000;68:1–8. 10914483

[13] Cheng Y, Ma XL, Wei YQ, et al. Potential roles and targeted therapy of the CXCLs/CXCR2 axis in cancer and inflammatory diseases. Biochim Biophys Acta. 2019;1871:289–312. 10.1016/j.bbcan.2019.01.005 30703432

[14] Feniger-Barish R, Belkin D, Zaslaver A, et al. GCP-2-induced internalization of IL-8 receptors: hierarchical relationships between GCP-2 and other ELR(+)-CXC chemokines and mechanisms regulating CXCR2 internalization and recycling. Blood. 2000;95(5):1551–1559. 10688807

[15] Waugh DJ, Wilson C. The interleukin-8 pathway in cancer. Clin Cancer Res. 2008;14:6735–6741. 18980965 10.1158/1078-0432.CCR-07-4843

[16] Keeley EC, Mehrad B, Strieter RM. CXC chemokines in cancer angiogenesis and metastases. Adv Cancer Res. 2010;106:91–111. 20399957 10.1016/S0065-230X(10)06003-3 PMC3069502

[17] Donahue TR, Hines OJ. CXCR2 and RET single nucleotide polymorphisms in pancreatic cancer. World J Surg. 2009;33:710–715. 19057948 10.1007/s00268-008-9826-z

[18] Liu Q, Li A, Tian Y, et al. The CXCL8-CXCR1/2 pathways in cancer. Cytokine Growth Factor Rev. 2016;31:61–71. 27578214 10.1016/j.cytogfr.2016.08.002 PMC6142815

[19] Matsuo Y, Takeyama H, Guha S. Cytokine network: new targeted therapy for pancreatic cancer. Curr Pharm Des. 2012;18:2416–2419. 22372505 10.2174/13816128112092416

[20] Li L, Xu L, Yan J, et al. CXCR2-CXCL1 axis is correlated with neutrophil infiltration and predicts a poor prognosis in hepatocellular carcinoma. J Exp Clin Cancer Res. 2015;34:129. 26503598 10.1186/s13046-015-0247-1 PMC4621872

[21] Pączek S, Łukaszewicz-Zając M, Gryko M, et al. The Clinical Utility of Serum CXCR-2 Assessment in Colorectal Cancer (CRC) Patients. Anticancer Res. 2021;41:1421–1428. 33788733 10.21873/anticanres.14899

[22] Presti M, Mazzon E, Basile MS, et al. Overexpression of macrophage migration inhibitory factor and functionally-related genes, D-DT, CD74, CD44, CXCR2 and CXCR4, in glioblastoma. Oncol Lett. 2018;16:2881–2886. 30127875 10.3892/ol.2018.8990 PMC6096183

[23] Strieter RM, Polverini PJ, Arenberg DA, et al. Role of C-X-C chemokines as regulators of angiogenesis in lung cancer. J Leukoc Biol. 1995;57:752–762. 7539029 10.1002/jlb.57.5.752

[24] Imafuji H, Matsuo Y, Ueda G, et al. Acquisition of gemcitabine resistance enhances angiogenesis via upregulation of IL‑8 production in pancreatic cancer. Oncol Rep. 2019;41:3508–3516. 31002348 10.3892/or.2019.7105

[25] Payne AS, Cornelius LA. The role of chemokines in melanoma tumor growth and metastasis. J Invest Dermatol. 2002;118:915–922. 12060384 10.1046/j.1523-1747.2002.01725.x

[26] An H, Xu L, Chang Y, et al. CXC chemokine receptor 2 is associated with postoperative recurrence and survival of patients with non-metastatic clear-cell renal cell carcinoma. Eur J Cancer. 2015;51:1953–1961 26188847 10.1016/j.ejca.2015.06.125

[27] Nishi T, Takeuchi H, Matsuda S, et al. CXCR2 expression and postoperative complications affect long-term survival in patients with esophageal cancer. World J Surg Oncol. 2015;13:232. 26231560 10.1186/s12957-015-0658-7 PMC4522106

[28] Maeda S, Kuboki S, Nojima H, et al. Duffy antigen receptor for chemokines (DARC) expressing in cancer cells inhibits tumor progression by suppressing CXCR2 signaling in human pancreatic ductal adenocarcinoma. Cytokine. 2017;95:12–21. 28214673 10.1016/j.cyto.2017.02.007

[29] Savage SA, Abnet CC, Mark SD, et al. Variants of the IL8 and IL8RB genes and risk for gastric cardia adenocarcinoma and esophageal squamous cell carcinoma. Cancer Epidemiol Biomarkers Prev. 2004;13:2251–2257. 15598788

[30] Brown EE, Fallin D, Ruczinski I, et al. Associations of classic Kaposi sarcoma with common variants in genes that modulate host immunity. Cancer Epidemiol Biomarkers Prev. 2006;15:926–934. 16702372 10.1158/1055-9965.EPI-05-0791

[31] Kamangar F, Abnet CC, Hutchinson AA, et al. Polymorphisms in inflammation-related genes and risk of gastric cancer (Finland). Cancer Causes Control. 2006;17:117–125. 16411061 10.1007/s10552-005-0439-7

[32] Lee KM, Shen M, Chapman RS, et al. Polymorphisms in immunoregulatory genes, smoky coal exposure and lung cancer risk in Xuan Wei, China. Carcinogenesis. 2007;28:1437–1441. 17361014 10.1093/carcin/bgm030

[33] Andrew AS, Gui J, Sanderson AC, et al. Bladder cancer SNP panel predicts susceptibility and survival. Hum Genet. 2009;125:527–539. 19252927 10.1007/s00439-009-0645-6 PMC2763504

[34] Snoussi K, Mahfoudh W, Bouaouina N, et al. Combined effects of IL-8 and CXCR2 gene polymorphisms on breast cancer susceptibility and aggressiveness. BMC Cancer. 2010;10:283. 20540789 10.1186/1471-2407-10-283 PMC2895614

[35] Bondurant KL, Lundgreen A, Herrick JS, et al. Interleukin genes and associations with colon and rectal cancer risk and overall survival. Int J Cancer. 2013;132:905–915. 22674296 10.1002/ijc.27660 PMC3470814

[36] Singh V, Jaiswal PK, Kapoor R, et al. Impact of chemokines CCR5∆32, CXCL12G801A, and CXCR2C1208T on bladder cancer susceptibility in north Indian population. Tumour Biol. 2014;35:4765–4772. 24430363 10.1007/s13277-014-1624-7

[37] Ryan BM, Robles AI, McClary AC, et al. Identification of a functional SNP in the 3ʹUTR of CXCR2 that is associated with reduced risk of lung cancer. Cancer Res. 2015;75:566–575. 25480945 10.1158/0008-5472.CAN-14-2101 PMC4315715

[38] Franz JM, Portela P, Salim PH, et al. CXCR2 +1208 CT genotype may predict earlier clinical stage at diagnosis in patients with prostate cancer. Cytokine. 2017;97:193–200. 28668699 10.1016/j.cyto.2017.06.001

[39] DerSimonian R, Laird N. Meta-analysis in clinical trials revisited. Contemp Clin Trials. 2015;45:139–145. 26343745 10.1016/j.cct.2015.09.002 PMC4639420

[40] Ng KH, Peh WC. Presenting the statistical results. Singapore Med J. 2009;50:11–14. 19224078

[41] Mootha VK, Lindgren CM, Eriksson KF, et al. PGC-1alpha-responsive genes involved in oxidative phosphorylation are coordinately downregulated in human diabetes. Nat Genet. 2003;34:267–273. 12808457 10.1038/ng1180

[42] Sun W, Shi H, Yuan Z, et al. Prognostic Value of Genes and Immune Infiltration in Prostate Tumor Microenvironment. Front Oncol. 2020;10:584055. 33194726 10.3389/fonc.2020.584055 PMC7662134

[43] Pan HY, Mi YY, Xu K, et al. Association of C-reactive protein (CRP) rs1205 and rs2808630 variants and risk of cancer. J Cell Physiol. 2020;235:8571–8584. 32329054 10.1002/jcp.29701

[44] Zhang LF, Xu K, Tang BW, et al. Association between SOD2 V16A variant and urological cancer risk. Aging (Albany NY). 2020;12:825–843. 31929112 10.18632/aging.102658 PMC6977677

[45] Gabellini C, Trisciuoglio D, Desideri M, et al. Functional activity of CXCL8 receptors, CXCR1 and CXCR2, on human malignant melanoma progression. Eur J Cancer. 2009;45:2618–2627. 19683430 10.1016/j.ejca.2009.07.007

[46] Kemp DM, Pidich A, Larijani M, et al. Ladarixin, a dual CXCR1/2 inhibitor, attenuates experimental melanomas harboring different molecular defects by affecting malignant cells and tumor microenvironment. Oncotarget. 2017;8:14428–14442. 28129639 10.18632/oncotarget.14803 PMC5362416

[47] Yang Y, Luo B, An Y, et al. Systematic review and meta-analysis of the prognostic value of CXCR2 in solid tumor patients. Oncotarget. 2017;8:109740–109751. 29312644 10.18632/oncotarget.22285 PMC5752557

[48] Qiao B, Luo W, Liu Y, et al. The prognostic value of CXC chemokine receptor 2 (CXCR2) in cancers: a meta-analysis. Oncotarget. 2018;9:15068–15076. 29599927 10.18632/oncotarget.23492 PMC5871098

[49] Wu H, Zhang Z, Xiao XY, et al. Toll-like receptor 2 (TLR2) is a candidate prognostic factor in testicular germ cell tumors as well as an indicator of immune function in the tumor microenvironment. Bioengineered. 2021;12:1939–1951. 34002664 10.1080/21655979.2021.1927560 PMC8806693

[50] Wu H, Zhang ZY, Zhang Z, et al. Prediction of bladder cancer outcome by identifying and validating a mutation-derived genomic instability-associated long noncoding RNA (lncRNA) signature. Bioengineered. 2021;12:1725–1738. 33955803 10.1080/21655979.2021.1924555 PMC8806732

